# Transactions of the Medical and Physical Society of Bombay

**Published:** 1839

**Authors:** 


### TRANSACTIONS OF THE MEDICAL AND PHYSICAL SOCIETY OF BOMBAY. Octavo, pp. 370. Bombay, 1838. J. M. Richardson, Cornhill.

We have, on a previous occasion, noticed very briefly this volume of Transactions. But the Surgeons of a Presidency like Bombay have some claims on the notice of their European brethren, and the latter must be curious to learn the current of thought, or feeling, or of occupation, that flows in the channel of professional life in India.

We quote from the Preface to the volume before us, a rather interesting passage. It exhibits in a new light the hackneyed and seemingly familiar subject of change of climate.

"Among other branches of research, which in an especial manner, require the attention of the medical observer between the tropics—may be instanced, the application of the facts ascertained by the experiments of MILNE EDWARDS on the influence *of physical agents on life,* to the explanation of the phenomena of disease.

The facts to which allusion is principally intended, are

1. The greater capacity possessed by warm-blooded animals, of generating heat in a cold, than in a warm climate, or season.

2. This change from great to small capacity of generating animal heat, is not sudden, but gradual, and consequently, circumstances may frequently arise, in which, the capacity will not be in relation to the necessity.

3. The capacity of generating animal heat, is in relation to the quantity of oxygen consumed, during respiration.

4. The amount of oxygen consumed, is also influenced by conditions of the animal in regard to diet, wakefulness, sleep, &c.

5. This relation of certain conditions of the animal, to the consumption of oxygen—establishes a relation between those same conditions, and the capacity of generating animal heat.

To enter into the detail which these facts naturally suggest, would be misplaced here: it is sufficient to call attention to the importance of their consideration, when directing change of climate: for it may with propriety be said, that this important instrument in the prevention, and cure of disease, though daily recommended, and had recourse to, is one, regarding which, we have not definite, or applicable information. Nor can it be advanced, that this state of ignorance on our part, is a necessary result of the abstruseness of the subject; till we shall have studied it, in strict relation to the present condition of meteorological science, and to that additional knowledge of the laws of life, which the experiments of Milne Edwards, have placed at our command.

It may be urged, that medical writers in colder latitudes, have written much, and well, on the subject of change of climate. But without entering into any consideration of the merits of these works, it may at once be presumed, that if, they are applicable to the conditions of *colder latitudes,* they cannot be so in any extent, to countries between the *tropics.*

Change from a cold climate, has always reference to recourse to a warmer one, and has an antiphlogistic tendency: whereas change from a tropical climate, has always reference to recourse to a colder one, and has a tonic tendency: *it is the difference between antiphlogistic regimen and the exhibition of tonics.*

In regard to the 3d, 4th, and 5th facts, it may be remarked, that if we regard the degree of oxygenation of the blood, as not confined in its influence, to the function of animal heat—but as extending to all functions, to the actions of which, the circulation of the blood is essential: then, to a very casual reflection, the wide range of their application will at once, be apparent." v.

The Secretary, Mr. Morehead, goes on to remark:—

"*The manner of rearing the children of Europeans in India, and the question of its propriety,* are also intimately associated with these physiological facts, and may be thus expressed.

1. By the modification of the action of what functions, are the characteristics of different periods of life, and different varieties of the human race, brought about?

2. Is it rational to suppose, that a knowledge of the laws of these modifications, may lead to a knowledge of the means, by which, their operation may be counteracted, when unfavourable, or invited when the reverse?

These propositions tend to a course of inquiry, as yet unentered upon, but of great importance, and falling within the province of the medical practitioner in India, in an especial manner, to attempt." vi.

Mr. Morehead indulges in some speculative observations, which we must also give in his own words.

"To attempt," he says, "to acquire a knowledge of the laws of disease, by in the first instance, studying the laws of external agencies, and in the second, their manner of operation on the animal system—would be, to revert to the direction cf research, followed by the FATHERS of our art, and neglected since the days of SYDENHAM and HUXHAM. It may be further objected, that this order of procedure, would be to desert that mode of inquiry of the modern schools—which seems to be, in a fair way of leading, to a juster knowledge of the laws of disease —which has reference to a knowledge of the ultimate phenomena of disease, *(morbid anatomy)* acquired in the hope of ascending, by gradations of generalization, to a knowledge of their initiatory laws.

That both are legitimate modes of inquiry, cannot be questioned. The mistake, perhaps, has been, in advocating the one, to the exclusion of the other, instead of allowing the circumstances of the inquirer, to dictate the preference. Where there is much variety in the phenomena of external agencies—of climate, season, modes of life, &c. as in India, then, to study the laws of these phenomena, in relation to their effects, ('the production of disease,') ought to occupy much attention. But when these external agencies are less operative, better understood, and guarded against, then, the mode of inquiry, from ultimate *individual* phenomena, to the higher stages of generalization, is that, which more naturally suggests itself.

If there be reason in nature for this distinction, it admits a preponderance to the importance of the study of morbid anatomy, in temperate climates; but does not make the same admission, in regard to the study of the phenomena of disease, between the tropics. The acknowledgment of such a principle, is of this further consequence, that it at once establishes the conviction, that, *to be slavish imitators of the doctrines, and systems of the European schools, is to shew, that we do not estimate our position here, at its true value to science.*

To revert again to these two manners of inquiry.

In arguing, the probability of failure on our part, from the want of success of SYDENHAM, and HUXHAM, it ought to be recollected—that HUXHAM did not know, the chemical constitution of the atmospheric air, and that since his time, important additions have been made, to our knowledge of the laws of life. To throw the lights derivable from the science of meteorology, and the experiments of MILNE EDWARDS, on this mode of inquiry, would be, entirely to alter its complexion. Whilst, without wishing to take from the value, of the study of *morbid anatomy*, it may be with reason suspected, that its importance is not always accurately measured—that it is too often considered, as the *end*, instead of the *means*.

It is perhaps occasionally forgotten, that the facts of *morbid anatomy*, are but a part of a series of phenomena—the knowledge of the whole of which, it is hoped, may in time lead, to a knowledge of the general laws of disease. Should a knowledge of these laws, ever teach a more efficient system of prevention and cure of disease—then it will be unnecessary, for the *future* medical philosopher, to go over the stages of observation by which, this knowledge has been reached: he may not in fact, enjoy the opportunity, because, if the object of studying the appearances of morbid anatomy, be, to lead to a better system of cure—then it follows, that if this object ever be gained, in any considerable degree, *the effect will be, to prevent the recurrence of those very appearances, which now constitute a department of study, under the name of morbid anatomy.*" xi.

It cannot, we think, be doubted, that morbid anatomy does not constitute the whole of pathology. The study of morbid anatomy is now acknowledged to be only the study of effects, the lesion being the result of prior morbid action. No one in his senses would counsel attention to morbid anatomy exclusively, although it must be owned that some persons who profess not to have lost their senses, virtually pursue this course. A sound acquaintance with morbid anatomy informs us of the tendencies of morbid action—its causes, and its treatment constitute separate subjects of investigation. It may be that our Indian brethren enjoy better opportunities than ourselves, for studying the causes of disease, or those external agencies which induce disease; yet we cannot perceive what their advantages are. We should say that the great field for cultivation at present, is the chemistry of the fluids in morbid conditions of the body.

The majority of the papers in these Transactions are not adapted for our pages. They refer to matters which peculiarly interest residents in India. But we may allude, as a subject of curiosity, to some Notes by Dr. Percival Lord, on the

### State of Medical and Surgical Science in the Countries bordering on the Indus.

It may be reasonably questioned whether we derive instruction from contemplating the state of science amongst barbarians. But some degree of amusement may be obtained in this manner, and perhaps the contrast between the arts of civilized and of uncivilized nations may conduce to our own information.

*A great Indian Lithotomist.*—Dr. Lord was so fortunate as to meet at Dera Ismael Khan, a man much renowned for his skill in lithotomy. He had no sound; his cutting instrument was a spear-shaped lancet, exceedingly rude in its construction. He ascertained the existence of the stone by introducing his finger into the rectum, and if necessary, pushing down the bladder from above.

He had operated, says Dr. Lord, on about two hundred cases, of which seven or eight were women: he could not say how many children. Of these about twenty, all adults, had died. Death never followed immediately after the operation from hæmorrhage, but, when it did occur, was always preceded by intumescence of the belly, sickness of stomach, &c. &c.; in other words, the patient died of the subsequent inflammation, and this he knew no means of checking.

Before proceeding to operate, he always insisted on the patient giving him a written certificate acquitting him of all responsibility, in case death should follow. The man was perfectly ignorant of anatomy, and proceeded in this simple manner, pretty much as our ancestors did before the days of Frere Jaques.

The patient was placed on the ground on his back, with his legs drawn up, and the knees held asunder by assistants. The operator introduced two fingers into the rectum, (one if the patient were a child,) and endeavoured to hook with them the stone, aiding himself by pressing down from above, with the other hand. When he had once hooked the stone, he stated, he was always certain of getting it out. For this purpose he pressed it down on the perinæum, making it project on one side or other, as its situation rendered convenient. He avoided the raphé, as, he said, cutting there destroyed the generative powers. Having made the stone protrude, he pushed in his spear-shaped lancet until the point met the stone, and then with the shoulder enlarged his opening, first upwards and then downwards, until the point which he moved along the stone was found to have reached its edge in both directions. In doing this, he employed considerable force.

The knife is now withdrawn, and two fingers introduced, with which the incision is stretched, and torn until it will admit his second instrument, which is neither more nor less than a large goat's, or antelope's horn. The point of this is entered above the stone, and the instrument caused to revolve, using the pubis as a fulcrum, and then, with the practitioner's whole strength, the horn, thus converted into a lever, is suddenly pushed back towards the patient's body, while the point revolving, of course, in the opposite direction, jerks out the stone through the aperture.

Such is the proceeding in all cases: the only difference made with women is, that the incision is commenced at rather a greater distance from the raphé; the finger in both cases being introduced into the rectum, never into the vagina. The only subsequent treatment is to rub the parts over with oil, or, if the patient be of the better class, an ointment composed of common salt, turmeric, and the impure carbonate of soda, got from the *lāna* or salt plant, is smeared on.

"The number of deaths, as stated, is surprisingly small, but I am inclined to think they have been very much under-rated. The man is very ignorant, keeps no register of any kind, and merely gives his numbers by guess, and in a vague manner. Still he is nearly consistent in his proportions, for on asking him, at a separate interview, how many operations his brother (who is also a surgeon) had performed and how many patients lost, he replied 40 operations and 5 deaths, adding, there might have been one or two more. His father, he said, had operated in cases without number, and many of his patients had come from the remotest parts of Khorasàn. He was not aware, whether he ever cut into the urethra or rectum, but some melancholy cases came before me in which, either from the operation or the subsequent formation of abscesses, both these opened into a common cavity, and dreadful ulceration and sloughing had supervened.

Stones, he said, were occasionally impacted in the urethra, and these he cut on, and removed without hesitation. I must add, in justice to my brother practitioner, that his scale of charges was extremely moderate: a poor man, he cut for stone for two rupees, a rich man, he said, usually presented him with a horse or a camel. But stone in the bladder is an object, as well of medical, as of surgical practice, and various mixtures are prescribed for the purpose of expelling the stone, and rendering the operation unnecessary. Most of these are rather likely to act as diuretics than solvents, still the alkali which they generally contain, may give them some of this latter quality also." 281.

*An Indian Operation for Cataract.*—But, continues Dr. Lord, he was also skilful in removing cataract, though his success, if he ever did succeed, was inexplicable on any principles known to us. Of the eye, and its contents, he had not the remotest idea. Cataract he called and considered *purdah* (a screen); to remove that screen, he announced, as the object of his operation, but conscious, that he had never really succeeded in withdrawing this screen, he usually, before the operation furnished himself with a small shred of onion peel, which he managed to wrap round his instrument, so that on withdrawing it from the eye, and plunging it into a vessel of water, the shred might appear floating on the surface, thus affording the most indubitable evidence of his skill and success.

His implements were certainly wretched. He had an old lancet with which he made his incision, and, to prevent it entering too far, had it wrapped round with cotton thread, to within about two lines of the top, so as to leave little more than was sufficient, for cutting through the thickness of the sclerotica. His incision was always made from below, directing the patient to look up, and entering the lancet over the lower eyelid into the white of the eye, when he had it well exposed.

He did not seem particular as to the exact spot; any where, between the cornea and the reflection of the conjunctiva, was all the same to him. Having made his incision, he withdrew the lancet, and proceeded to introduce a long copper probe, on the top of which, was a sort of irregular, triangular pyramid blunted at the point, to prevent it, as he said, doing mischief.

Now commenced the serious business of the operation, for taking the handle of the probe in his fingers, he set to, twisting it round and round until, by some chance, one of the corners hitched against the lens, and drew it out of the axis of vision. In this process great part of the vitreous humour was discharged, dreadful laceration occurred, and, not unfrequently, blood flowed freely.

The minute the patient exclaimed, he saw, the instrument was withdrawn, (the lens being never extracted, but merely reclined, or depressed,) a piece of cotton dipped in a mixture of opium, oil and turmeric was dabbed on the eye, and the operation declared to be finished. Occasionally the additional precaution was taken, of making three issues with a hot iron, one behind, and two over the eyes; but no further treatment was known or thought of. It will readily be believed that, after such operations, the occurrence of inflammation of the globe is almost inevitable. This they have no means of checking, and Dr. Lord believes that the operation ends far more frequently in the total destruction of the globe, than in the restoration of even imperfect vision.

*Studies of an Indian Physician.*—" Of their medical art it scarce becomes me to speak after a transient observation of a few months, as a venerable practitioner at Pind Mulik Oulea, in reply to my enquiries on the subject, told me that he had spent thirteen years learning his profession, before he ever ventured on a single prescription. I was however a little re-assured when I found he had employed his whole time in committing to memory four books; viz. first, the Jumguntuk, which gives a history of the structure of the body; second, the Kok-Shaster, which treats of the diseases of females, and other matters connected with the sex; third, the Bèd-Munùrut, which gives prescriptions; and last, the Nidàn, which teaches the different forms of disease.

As a specimen of what he had learned, he told me that there were 18 kinds of gonorrhœa, and 84 of belly-aches. I was astonished at this abundance, but was informed that the variety was not in the nature, but the situation of the pain, the abdomen being divided into so many distinct regions.

Further, to impress me with an idea of his anatomical skill, he informed me that the arm contained three principal veins, namely, Budsalìk, which went to the eyes, and was to be opened in all diseases of the head; Sir-ì-rù, which, having its source in the liver and heart, was to be opened in cases of jaundice, palpitation, &c.; and Haftandàm, which went, he did not exactly know where, but which was to be opened for rheumatism only. A Mùltan practitioner had recounted to me seven veins, but, not having taken them down at the time, I am unable to report on the remaining four." 286.

*A grand Aphrodisiac.*—"Mixtures of this kind are considered the triumphs of the art, and for these, practitioners receive the highest rewards; it is therefore, perhaps, sufficiently natural, that they should have engaged so much of their attention. In proportion to the high demand for them, is the difficulty of obtaining an explanation of their contents, nor was it without considerable persuasion, and the gift of, I know not, how many empty bottles, (here a highly-prized present,) that I succeeded in getting the following, an undoubted original, composed expressly for the Nuwab, who ruled the Deraját previous to the Sikh invasion, and the birth of whose child was attributed, both by himself and his courtiers, to its potent influence.

Take of Shingruf, (cinnabar,) 1½ tola.

Tie it up in a cloth, and put it in a vessel containing a maund and half of milk. Boil slowly, until all the milk is boiled away. (By this proceeding the Shingruf is supposed to attract to, and concentrate in itself the whole nutritive qualities of the milk.) Then place it in ¾ maund of onion water, and repeat the same process. (By this it is supposed to acquire the heating qualities of the onion.) Then take purified opium 4½ tolas; dissolve in water, and boil Shingruf a third time in this water. (By this it becomes intensely stimulant.)

But now comes the grand invention.

Take five chickens, bray them to a soft paste in an iron mortar, picking out all the bones; add as much bilâwa (marking-nut) as shall equal the weight of the flesh, mix intimately, and divide all this into ten parts.

In one of these parts place the Shingruf, wrapping it up as in a ball; cover it over with flour and fry it in ghee, until the ball becomes thoroughly browned. Then take out the Shingruf, transfer it to another ball, and fry, and so on, till the Shingruf has been fried in all ten parts successively. The balls may now be thrown away, and the Shingruf is fit for use. For this purpose reduce it to powder. The dose is the weight of one grain of rice, mixed with butter, and washed down with one seer of milk.

This the practitioner told me, he had prepared for the Nuwab, as the most powerful of all aphrodisiacs, and added that he had kept a little dose for himself, the efficacy of which perfectly astonished him." 291.

*Arsenic given for Ague.*—"The preparations of arsenic are generally used only as external applications, but I was astonished to find that the *Zurd Sunkiah*, yellow sulphuret of arsenic, was sometimes employed in small doses (they said *half rutty*) for obstinate cases of intermittent fever. I certainly was not prepared to find Dr. Fowler's specific, brought into practical operation in the Derajàt." 293.

### II. THE MANNER OF BREEDING LEECHES PRACTISED IN THE DECCAN.

Mr. Gibson communicates this in a letter addressed to the Secretary of the Society. In consequence of information which he had received, Mr. Gibson proceeded to Sowla, about eight miles from Seroor.

"I am happy to say," he writes, "that I found the particulars mentioned in your letter, generally verified. The leeches had been taken from their cocoons this morning, and therefore we could not see them, during the actual process of transition, but we saw the parent leeches; and I have the pleasure to inclose, a parcel of the cocoons from which the young were taken, and to add, that we saw the young brood in full activity and life, each leech being in length three quarters of an inch.

The people were very reluctant to show us the process of production in actual operation, but at length, on promising a reward, and by other persuasive means,

We succeeded in inducing them to do so. We were taken into an inner apartment, and a large earthen vessel buried in the earth, and filled with brownish soil, apparently without any admixture, and most carefully covered over, was shown to us. On raising the covering, we perceived, that the leeches had all made their way to the lower part of the vessel, leaving the upper parts of the earth furrowed, and perforated with their tracks.

The people told us, that the leech, on reaching maturity, is fed on a buffalo, or, if opportunity offers, on a human subject; it is then put into the earthen vessel to breed, but nothing is known of any distinction of sexes.

In this vessel it remained, in hot weather, from one to one and a half months, and in the cold weather from one and a half to two months. The vessel is then opened, the leeches are taken out, and to each is found attached the membranous reticulated egg, herewith sent, and this egg contains from four to six leeches. The young, on being taken out, are put into an open earthen vessel filled with brownish earth, and after two months, are said to be fit for use; if not then used on the human subject, they are fed on buffaloes, and afterwards placed to breed.

The people say, that the animal generally lives four, sometimes five, and rarely six months. They describe this process of production, as being carried on at Sowla, at Bela, at Chap Maroli, at Sungunmair, but, to their knowledge, at no other places; and that it is altogether in the hands of a few Musulman Bungias, who are probably relations. It seems not to be general throughout the country.

I need not advert to the useful purposes to which this art may be applied, in breeding leeches in those countries where they are scarce, on shipboard, or in England, where they are frequently sold at most exorbitant prices." 314.

### III. Fatal Rupture of the Spleen from slight Violence.

Four cases of death from ruptured spleen are reported by Mr. Heddle amongst some other instances of violent death. We shall quote one, sufficient for our purpose, as it shews the slight degree of violence which in certain conditions of the spleen, as of the liver, may occasion laceration of the organ.

*Case.*—The subject of this was a Portuguese cook, who was attached to the 2nd Battalion of Artillery. On the morning in which he died, deceased, who was a middle-aged man, was observed fighting with a little boy not more than 12 or 14 years of age, and who in stature scarcely reached to the deceased's waist. Shortly after, the fight terminated by the little boy making his escape, and running into the bazar, the deceased became very weak and faint, and complained of uneasiness of the left side: these symptoms increased, and in a few minutes he expired.

On examining the body externally, no mark of injury could be detected. The abdomen was distended, and on making an incision through the parietes, a large accumulation of fluid blood was observed, filling the cavity of the peritoneum.

The abdominal viscera, when examined, proved to be in general healthy; the spleen alone being in a diseased state, much enlarged, and so tender that the slightest effort sufficed to push the end of the finger into its substance. This organ presented a linear laceration across the surface, which extended to the depth of half an inch. From the vessels ruptured by this lesion, had proceeded the hæmorrhage which proved fatal.

It was stated, that the deceased had previously been subject to fever, and had only recently returned from Socotra, whither he had accompanied a detachment of the troops, that suffered so severely from sickness on that island.

It was not clear whether the exertions of the deceased himself, or any blow inflicted by the fist (for the little boy had no stick, or weapon of any kind in his hand) of his adversary, caused the rupture.

We trust that the Bombay Society will persevere with the publication of their Transactions, and that each succeeding volume will bear more flattering evidence of their zeal, in the cause of the promotion of medical science.

